# Racial differences in real-world outcomes of first-line therapies for advanced renal cell carcinoma

**DOI:** 10.1093/oncolo/oyae354

**Published:** 2024-12-19

**Authors:** Daniel M Geynisman, William S John, Taavy A Miller, Parisa Asgarisabet, Sarah B Guttenplan, Xin Yin, Kristin M Zimmerman Savill, Lindsay McAllister, Lisa Rosenblatt

**Affiliations:** Department of Hematology/Oncology, Fox Chase Cancer Center, Philadelphia, PA 19111, United States; Real-World Evidence and Insights, Cardinal Health, Dublin, OH 43017, United States; Real-World Evidence and Insights, Cardinal Health, Dublin, OH 43017, United States; Real-World Evidence and Insights, Cardinal Health, Dublin, OH 43017, United States; Global Health Economics and Outcomes Research, Bristol Myers Squibb, Princeton, NJ 08648, United States; Global Health Economics and Outcomes Research, Bristol Myers Squibb, Princeton, NJ 08648, United States; Real-World Evidence and Insights, Cardinal Health, Dublin, OH 43017, United States; Real-World Evidence and Insights, Cardinal Health, Dublin, OH 43017, United States; Global Health Economics and Outcomes Research, Bristol Myers Squibb, Princeton, NJ 08648, United States

**Keywords:** renal cell carcinoma, ethnic and racial minorities, nivolumab, pembrolizumab, tyrosine kinase inhibitors

## Abstract

**Background:**

Given the historical underrepresentation of racial minorities in clinical trials, little is known about racial differences in outcomes of first-line therapies for advanced renal cell carcinoma (aRCC). This study described patient characteristics and clinical outcomes of first-line therapies for aRCC, including nivolumab + ipilimumab, pembrolizumab + axitinib, and tyrosine kinase inhibitors, by race in the real-world setting.

**Methods:**

We conducted a retrospective medical chart review of patients with intermediate/poor-risk clear-cell aRCC. Treating physicians abstracted patient data from electronic medical charts.

**Results:**

Among 346 patients with intermediate/poor-risk aRCC, a higher proportion of African-American/Black (*n* = 78) versus White (*n* = 268) patients had poorer baseline functional performance (ECOG-PS ≥ 2: 37.2% versus 21.3%). African-American/Black patients trended toward numerically lower overall response rates and shorter overall survival for nivolumab + ipilimumab or pembrolizumab + axitinib than White patients.

**Conclusions:**

These findings provide important insights into racial differences in first-line aRCC treatment outcomes within real-world settings. Additional research with larger sample sizes is warranted.

## Introduction

Historically, African-American/Black patients have been underrepresented in clinical trials for advanced renal cell carcinoma (aRCC) (eg, 1.2% among CheckMate 214 participants^1^; 2.1% among KEYNOTE-426 participants^2^). Incidence rates of aRCC, however, are increasing among this population.^3,4^ Little is known about racial differences in the use of first-line therapies and subsequent clinical outcomes in the real-world setting. The aim of this study was to describe racial differences in demographic and clinical characteristics, adverse events (AEs), and clinical outcomes among patients with intermediate or poor (I/P)-risk clear-cell aRCC who initiated first-line treatment with current standard-of-care options including nivolumab plus ipilimumab (NIVO + IPI), pembrolizumab plus axitinib (PEM + AXI), or tyrosine kinase inhibitor (TKI) monotherapy.

## Methods

This was a non-interventional, retrospective, medical chart review study. Treating oncologists from the Cardinal Health Oncology Provider Extended Network abstracted de-identified patient-level data into an electronic case report form for eligible patients. The first phase of data collection was from February 12, 2021 to May 14, 2021, followed by an extended follow-up data collection among the same patient cohort from May 27, 2022 to June 29, 2022.

Eligible patients were ≥18 years of age at the time of aRCC diagnosis with clear-cell histology and received first-line therapy using NIVO + IPI, PEM + AXI, or a TKI (sunitinib, pazopanib, or cabozantinib) as a single agent. First-line therapy must have been initiated on or after May 01, 2018 for the NIVO + IPI or TKI monotherapy groups and on or after May 01, 2019 for the PEM + AXI group (index dates based on respective FDA approval dates for aRCC). Patients were required to be followed for at least 1 cycle (3-4 weeks) of therapy. Patients were excluded if they received one of the first-line therapies of interest as part of a clinical trial or had any active malignancy in the 3 years prior to initiation of first-line therapy. This analysis only included patients in the sample who were White or African-American/Black with I/P risk.

Demographic and clinical characteristics were summarized using descriptive statistics. Outcomes assessed included duration of therapy, real-world overall response rate (rwORR), real-world progression-free survival (rwPFS), overall survival (OS), and select AEs (based on expected immune-related AEs from IO regimens).^1,2,5^ Analyses were conducted using Statistical Analysis Software (SAS v9.4).

## Results

Among an overall sample of 346 aRCC patients with I/P risk, 184 received NIVO + IPI, 95 received PEM + AXI, and 67 received TKI monotherapy. A numerically higher proportion of African-American/Black (*n* = 78) versus White (*n* = 278) patients were male (73.1% vs 59.7%), had Medicare insurance (62.8% vs 51.5%), and had poor-risk status (50% vs 39.9%) and ECOG-PS ≥2 (37.2% versus 21.3%) at therapy initiation (Table 1). The median duration of follow-up from initiation of first-line therapy was 21.2 months for African-American/Black patients and 20.6 months for White patients.

**Table 1.** Baseline demographic and clinical characteristics stratified by race and first-line treatment regimen among patients with I/P-risk aRCC.

	Overall (*N* = 346)		1L NIVO+IPI (*n* = 184)		1L PEM + AXI (*n* = 95)		1L Single-agent TKI (*n* = 67)	
	Black/African-American	White	Black/African-American	White	Black/African-American	White	Black/African-American	White
	*n* = 78	*n* = 268	*n* = 34	*n* = 150	*n* = 28	*n* = 67	*n* = 16	*n* = 51
Sex at birth, male, *n* (%)	57 (73.1)	160 (59.7)	24 (70.6)	92 (61.3)	23 (82.1)	40 (59.7)	10 (62.5)	28 (54.9)
Median age at 1L initiation, years (range)	67.9 (45–83.1)	65.5 (39.6–88.5)	61.9 (45–75.2)	64 (40.4–81.9)	70.3 (54.2–79.4)	67.3 (39.6–83.4)	74 (58.6–83.1)	71.2 (46.8–88.5)
Payer at initiation of 1L therapy for aRCC, *n* (%)								
Medicare	49 (62.8)	138 (51.5)	14 (41.2)	65 (43.3)	23 (82.1)	35 (52.2)	12 (75.0)	38 (74.5)
Medicaid	17 (21.8)	21 (7.8)	7 (20.6)	12 (8.0)	8 (28.6)	6 (9.0)	2 (12.5)	3 (5.9)
Commercial	26 (33.3)	123 (45.9)	17 (50.0)	81 (54.0)	5 (17.9)	29 (43.3)	4 (25.0)	13 (25.5)
Military health insurance	0 (0.0)	1 (0.4)	0 (0.0)	0 (0.0)	0 (0.0)	0 (0.0)	0 (0.0)	1 (2.0)
Self-pay	2 (2.6)	0 (0.0)	1 (2.9)	0 (0.0)	1 (3.6)	0 (0.0)	0 (0.0)	0 (0.0)
Patient region of residence, *n* (%)								
Northeast	32 (41.0)	55 (20.5)	16 (47.1)	42 (28.0)	10 (35.7)	11 (16.4)	6 (37.5)	2 (3.9)
Midwest	7 (9.0)	60 (22.4)	4 (11.8)	31 (20.7)	0 (0.0)	12 (17.9)	3 (18.8)	17 (33.3)
South	26 (33.3)	71 (26.5)	9 (26.5)	35 (23.3)	12 (42.9)	21 (31.3)	5 (31.3)	15 (29.4)
West	13 (16.7)	82 (30.6)	5 (14.7)	42 (28.0)	6 (21.4)	23 (34.3)	2 (12.5)	17 (33.3)
Total observed lines of therapy for aRCC, *n* (%)								
1	43 (55.1)	146 (54.5)	18 (52.9)	80 (53.3)	14 (50.0)	41 (61.2)	11 (68.8)	25 (49.0)
2	27 (34.6)	89 (33.2)	10 (29.4)	51 (34.0)	13 (46.4)	18 (26.9)	4 (25.0)	20 (39.2)
≥3	8 (10.3)	33 (12.3)	6 (17.6)	19 (12.7)	1 (3.6)	8 (11.9)	1 (6.3)	6 (11.8)
1L therapy prescribed, *n* (%)								
Cabozantinib	4 (5.1)	20 (7.5)	0 (0.0)	0 (0.0)	0 (0.0)	0 (0.0)	4 (25.0)	20 (39.2)
NIVO + IPI	34 (43.6)	150 (56.0)	34 (100.0)	150 (100.0)	0 (0.0)	0 (0.0)	0 (0.0)	0 (0.0)
Pazopanib	7 (9.0)	26 (9.7)	0 (0.0)	0 (0.0)	0 (0.0)	0 (0.0)	7 (43.8)	26 (51.0)
PEM + AXI	28 (35.9)	67 (25.0)	0 (0.0)	0 (0.0)	28 (100.0)	67 (100.0)	0 (0.0)	0 (0.0)
Sunitinib	5 (6.4)	5 (1.9)	0 (0.0)	0 (0.0)	0 (0.0)	0 (0.0)	5 (31.3)	5 (9.8)
Risk categorization, *n* (%)								
Intermediate	39 (50.0)	161 (60.1)	11 (32.4)	85 (56.7)	18 (64.3)	41 (61.2)	10 (62.5)	35 (68.6)
Poor	39 (50.0)	107 (39.9)	23 (67.6)	65 (43.3)	10 (35.7)	26 (38.8)	6 (37.5)	16 (31.4)
Disease stage at initial dx, *n* (%)								
Stages I–III	17 (21.8)	56 (20.9)	9 (26.5)	26 (17.3)	5 (17.9)	13 (19.4)	3 (18.8)	17 (33.3)
Stage IV	60 (76.9)	211 (78.7)	25 (73.5)	123 (82.0)	22 (78.6)	54 (80.6)	13 (81.3)	34 (66.7)
Unknown	1 (1.3)	1 (0.4)	0 (0.0)	1 (0.7)	1 (3.6)	0 (0.0)	0 (0.0)	0 (0.0)
ECOG-PS at initiation of 1L therapy, *n* (%)								
0 or 1	49 (62.8)	211 (78.7)	27 (79.4)	134 (89.3)	19 (67.9)	50 (74.6)	3 (18.8)	27 (52.9)
2+	29 (37.2)	57 (21.3)	7 (20.6)	16 (10.7)	9 (32.1)	17 (25.4)	13 (81.3)	24 (47.1)
Median duration of follow-up from initiation of 1L therapy, months (range)	21.2 (2.9–46.8)	20.6 (1.0–48.1)	23.3 (3.9–46.8)	24.2 (2.2–47.5)	20.3 (3.4–36.2)	17.8 (1.0–33.7)	15.9 (2.9–40.2)	18.4 (1.2-48.1)
Status at last follow-up, *n* (%)								
Alive	39 (50.0)	152 (56.7)	18 (52.9)	95 (63.3)	13 (46.4)	35 (52.2)	8 (50.0)	22 (43.1)
Receiving no active therapy or palliative care	2 (2.6)	19 (7.1)	2 (5.9)	14 (9.3)	0 (0.0)	3 (4.5)	0 (0.0)	2 (3.9)
Currently receiving 1L therapy	14 (17.9)	57 (21.3)	6 (17.6)	36 (24.0)	4 (14.3)	13 (19.4)	4 (25.0)	8 (15.7)
Currently receiving 2L therapy	17 (21.8)	44 (16.4)	5 (14.7)	29 (19.3)	9 (32.1)	8 (11.9)	3 (18.8)	7 (13.7)
Currently receiving ≥ 3L therapy	4 (5.1)	17 (6.3)	3 (8.8)	9 (6.0)	0 (0.0)	6 (9.0)	1 (6.3)	2 (3.9)
Receiving palliative care only	0 (0.0)	4 (1.5)	0 (0.0)	1 (0.7)	0 (0.0)	2 (3.0)	0 (0.0)	1 (2.0)
Referred to hospice	0 (0.0)	2 (0.7)	0 (0.0)	2 (1.3)	0 (0.0)	0 (0.0)	0 (0.0)	0 (0.0)
Unknown, lost to follow-up	0 (0.0)	1 (0.4)	0 (0.0)	0 (0.0)	0 (0.0)	0 (0.0)	0 (0.0)	1 (2.0)
Other	2 (2.6)	8 (3.0)	2 (5.9)	4 (2.7)	0 (0.0)	3 (4.5)	0 (0.0)	1 (2.0)
Deceased	39 (50.0)	116 (43.3)	16 (47.1)	55 (36.7)	15 (53.6)	32 (47.8)	8 (50.0)	29 (56.9)

Abbreviations: 1L, first-line; aRCC, advanced renal cell carcinoma; CI, confidence interval; CR, complete response; dx, diagnosis; Eastern Cooperative Oncology Group performance status; KM, Kaplan-Meier; min, minimum; max, maximum; p25–p75, 25th and 75th percentiles; PD, progressive disease; PR, partial response; SD, stable disease; STD, standard deviation; TKI, tyrosine kinase inhibitor.

Percentages may not sum to 100.0% (or specified column total) due to rounding.

The proportion who discontinued first-line therapy and the duration of first-line treatment was similar by race (Table 2). There was a trend toward numerically lower rwORR in African-American/Black versus White patients for each 1L therapy (NIVO + IPI: 67.6% vs 77.3%; PEM + AXI: 64.3% vs 71.6%; TKI monotherapy: 50.0% vs 52.9%). Among African-American/Black versus White patients, the median PFS was numerically shorter for NIVO + IPI (14.9 vs 17.2 months), yet similar or longer for PEM + AXI (14.5 vs 14.9 months) and TKI monotherapy (15.5 vs 11.5 months), respectively, and there was a trend toward shorter median OS for each 1L therapy (NIVO + IPI: 27.5 vs 39.3 months; PEM + AXI: 22.6 vs 29.3 months; TKI monotherapy: 23.9 vs 26.3 months).

**Table 2.** Clinical outcomes stratified by race and first-line treatment regimen among patients with I/P-risk aRCC.

	Overall (*N* = 346)		1L NIVO + IPI (*n* = 184)		1L PEM + AXI (*n* = 95)		1L single-agent TKI (*n* = 67)	
	African-American/Black	White	African-American/Black	White	African-American/Black	White	African-American/Black	White
	*n* = 78	*n* = 268	*n* = 34	*n* = 150	*n* = 28	*n* = 67	*n* = 16	*n* = 51
Permanently discontinued 1L therapy, yes, *n*, (%)	64 (82.1)	209 (78.0)	28 (82.4)	114 (76.0)	24 (85.7)	53 (79.1)	12 (75.0)	42 (82.4)
Reasons for 1L discontinuation, *n* (%)^a^								
Disease progression	47 (60.3)	149 (55.6)	18 (52.9)	78 (52.0)	20 (71.4)	38 (56.7)	9 (56.3)	33 (64.7)
Scheduled duration of therapy completed	4 (5.1)	8 (3.0)	4 (11.8)	7 (4.7)	0 (0.0)	0 (0.0)	0 (0.0)	1 (2.0)
AE/toxicity/intolerability	2 (2.6)	7 (2.6)	2 (5.9)	5 (3.3)	0 (0.0)	2 (3.0)	0 (0.0)	0 (0.0)
Patient choice	4 (5.1)	24 (9.0)	1 (2.9)	15 (10.0)	1 (3.6)	4 (6.0)	2 (12.5)	5 (9.8)
Insurance/Financial issue	0 (0.0)	2 (0.7)	0 (0.0)	1 (0.7)	0 (0.0)	1 (1.5)	0 (0.0)	0 (0.0)
Death	5 (6.4)	16 (6.0)	3 (8.8)	6 (4.0)	2 (7.1)	7 (10.4)	0 (0.0)	3 (5.9)
Other	2 (2.6)	3 (1.1)	0 (0.0)	2 (1.3)	1 (3.6)	1 (1.5)	1 (6.3)	0 (0.0)
Median duration of 1L therapy (KM method), months^b^ (95% CI)	14.3 (11.5–16.2)	14.9 (13.7–15.7)	14.3 (7.0–16.7)	15.5 (14.7–17.5)	14.5 (7.1–20.0)	14.7 (10.5–17.1)	15.3 (8.9–31.2)	11.6 (7.2–13.7)
Best response to therapy, *n* (%)^c^								
CR	9 (11.5)	39 (14.6)	7 (20.6)	29 (19.3)	1 (3.6)	5 (7.5)	1 (6.3)	5 (9.8)
PR	40 (51.3)	152 (56.7)	16 (47.1)	87 (58.0)	17 (60.7)	43 (64.2)	7 (43.8)	22 (43.1)
SD	15 (19.2)	42 (15.7)	4 (11.8)	17 (11.3)	6 (21.4)	10 (14.9)	5 (31.3)	15 (29.4)
PD	12 (15.4)	28 (10.4)	6 (17.6)	13 (8.7)	4 (14.3)	8 (11.9)	2 (12.5)	7 (13.7)
Overall response rate (CR + PR), % (95% CI)	62.8 (51.5–74.1)	71.3 (65.7–76.9)	67.6 (50.5–84.8)	77.3 (70.3–84.4)	64.3 (44.8–83.8)	71.6 (60.1–83.2)	50.0 (22.4–77.6)	52.9 (38.3–67.6)
PFS from initiation of 1L (KM method), months^d^								
*N* of events, *n* (%)	59 (75.6)	182 (67.9)	23 (67.7)	94 (62.7)	24 (85.7)	48 (71.6)	12 (75.0)	40 (78.4)
Median, (95% CI)	14.9 (13.1–16.6)	15.7 (14.7–17.2)	14.9 (12.2–19.6)	17.2 (15.3–20.4)	14.5 (7.1–20.3)	14.9 (11.7–18.7)	15.5 (8.9–31.2)	11.5 (7.3–13.6)
PFS from initiation of 1L, % (95% CI)								
6 months	77.7 (66.6–85.5)	86.3 (81.5–89.9)	78.1 (59.5–88.9)	89.8 (83.6–93.7)	75.0 (54.6–87.2)	86.2 (75.1–92.6)	81.3 (52.5–93.5)	76.5 (62.3–85.9)
9 months	72.4 (60.9–81.1)	75.5 (69.8–80.3)	75.0 (56.2–86.6)	81.5 (74.2–86.9)	67.9 (47.3–81.8)	75.2 (62.7–84.0)	75.0 (46.3–89.8)	58.8 (44.1–70.9)
12 months	65.8 (54.0–75.3)	65.8 (59.7–71.2)	71.9 (52.9–84.3)	74.5 (66.6–80.8)	57.1 (37.1–72.9)	61.1 (48.1–71.8)	68.8 (40.5–85.6)	47.1 (33.0–59.9)
18 months	36.6 (25.7–47.5)	41.5 (35.2–47.6)	34.0 (17.9–50.9)	46.0 (37.3–54.2)	35.7 (18.9–53.0)	40.1 (28.0–51.9)	42.2 (18.1–64.6)	29.8 (17.5–43.0)
OS from initiation of 1L (KM method), months^e^								
*N* of events, *n* (%)	39 (50.0)	116 (43.3)	16 (47.1)	55 (36.7)	15 (53.6)	32 (47.8)	8 (50.0)	29 (56.9)
Median (95% CI)	27.0 (18.1–NE)	33.3 (29.3–39.3)	27.5 (16.0–NE)	39.3 (30.3–NR)	22.6 (11.9–NE)	29.3 (17.2–33.7)	23.9 (11.9–NE)	26.3 (17.6–38.2)
OS from initiation of 1L, % (95% CI)								
6 months	89.7 (80.5–94.7)	92.5 (88.7–95.1)	91.2 (75.1–97.1)	96.0 (91.3–98.2)	85.7 (66.3–94.4)	89.5 (79.2–94.8)	93.8 (63.2–99.1)	86.3 (73.4–93.2)
9 months	82.1 (71.6–89.0)	88.4 (83.9–91.7)	82.4 (64.9–91.7)	94.0 (88.8–96.8)	78.6 (58.4–89.8)	84.9 (73.8–91.6)	87.5 (58.6–96.7)	76.5 (62.3–85.9)
12 months	73.1 (61.8–81.5)	81.6 (76.4–85.8)	79.4 (61.6–89.6)	87.3 (80.8–91.7)	64.3 (43.8–78.9)	75.8 (63.6–84.4)	75.0 (46.3–89.8)	72.5 (58.1–82.7)
18 months	62.7 (50.9–72.4)	71.0 (65.1–76.1)	67.6 (49.2–80.6)	78.9 (71.3–84.7)	60.7 (40.4–76.0)	59.8 (46.7–70.6)	55.6 (28.6–75.9)	62.2 (47.3–74.0)
Any AE during 1L, yes, *n* (%)^f^	14 (17.9)	79 (29.5)	9 (26.5)	48 (32.0)	5 (17.9)	17 (25.4)	0 (0.0)	14 (27.5)
95% CI	[8.8–27.1]	[23.8–35.1]	[10.2–42.8]	[24.2–39.8]	[1.9–33.8]	[14.2–36.5]	[0.0–0.0]	[14.2–40.7]
Number of AE during 1L, *n* (%)								
0	64 (82.1)	189 (70.5)	25 (73.5)	102 (68.0)	23 (82.1)	50 (74.6)	16 (100.0)	37 (72.5)
1	8 (10.3)	58 (21.6)	6 (17.6)	35 (23.3)	2 (7.1)	14 (20.9)	0 (0.0)	9 (17.6)
2	6 (7.7)	20 (7.5)	3 (8.8)	12 (8.0)	3 (10.7)	3 (4.5)	0 (0.0)	5 (9.8)
3	0 (0.0)	1 (0.4)	0 (0.0)	1 (0.7)	0 (0.0)	0 (0.0)	0 (0.0)	0 (0.0)
Time to onset of first AE during 1L among those with an AE, months								
Median (range)	2.3 (0–33.1)	3.6 (0–44.1)	5.1 (0–33.1)	3.2 (0–31.1)	2.3 (1–14.1)	4.1 (0.6–26)	0 (0–0)	20.2 (1–44.1)

Abbreviations: 1L, first line; CI, confidence interval; CR, complete response; Eastern Cooperative Oncology Group performance status; KM, Kaplan-Meier; min, minimum; max, maximum; OS, overall survival; p25–p75, 25th and 75th percentiles; PD, progressive disease; PFS, progression-free survival; PR, partial response; SD, stable disease; STD, standard deviation; TKI, tyrosine kinase inhibitor.

Percentages may not sum to 100.0% (or specified column total) due to rounding.

^a^For combination therapies, reason for discontinuation is measured by the agent. If reason for discontinuation for NIVO and IPI are different, NIVO is reported. If reason for discontinuation for PEM and AXI are different, PEM is reported.

^b^Defined as the time from initiation to discontinuation of a given line of therapy for any reason; patients who had not discontinued the given line of therapy by the time of data collection were censored at the date of last encounter.

^c^If best response was provided in extended follow-up, that value is reported; however, if the best response from extended follow-up was missing, the best response from the first phase of data collection is reported.

^d^Time from initiation of 1L therapy to charted disease progression based on radiographic imaging or death from any cause, whichever occurred first. Patients who discontinued 1L therapy for a reason other than progression or death were censored at discontinuation date. Patients still receiving 1L therapy at last encounter were censored on date of last encounter. Patients who did not progress during 1L, discontinued 1L, received an additional therapy, then died were censored at 1L discontinuation.

^e^Time from initiation of 1L therapy to death from any cause. Patients still alive at the last encounter were censored at the date of last encounter.

^f^Selected AE/toxicities are self-reported by the provider; the provider may have indicated up to 5 AEs/ toxicities during 1L. Categories of response are not mutually exclusive; the total may sum to more than the column total. If a patient had any AEs during the first phase of data collection, additional AEs were not assessed in extended follow-up data collection; extended follow-up only collected data on AEs for patients with no AEs reported during the first phase of data collection.

The rate of AEs in African-American/Black patients versus White patients was 26.5% vs 32.0% for NIVO + IPI, 17.9% vs 25.4% for PEM + AXI, and 0% vs 27.5% for TKI monotherapy (Table 2). The most common AE was rash during NIVO + IPI (35.1% of AEs), colitis during PEM + AXI (28.6% of AEs), and colitis (21.1% of AEs) during TKI monotherapy (Table S1).

## Discussion/Conclusions

In this real-world study of aRCC patients with I/P risk, we examined demographic and clinical characteristics, select AEs, and clinical outcomes of patients treated with first-line NIVO + IPI, PEM + AXI, or TKI monotherapy by race. We found that a numerically higher proportion of African-American/Black patients in the sample were male, and had Medicare insurance, poorer baseline functional status, and poor-risk status relative to White patients. Rates of AEs tended to be numerically lower among African-American/Black patients, especially for TKI monotherapy. Regarding clinical outcomes, African-American/Black patients trended toward numerically lower rwORR and shorter OS than White patients across first-line treatments; largest numerical differences were observed in rwORR and median OS for NIVO + IPI and PEM + AXI. These differences may be explained by higher rates of adverse baseline clinical characteristics, as well as lower rates of immune-related AEs, which have been associated with worse response to IO therapies,^5^ among African-American/Black patients than White patients. These findings are consistent with other studies showing a trend toward worse survival outcomes among African-American/Black vs White patients with aRCC treated with IO therapies or TKI monotherapies.^6,7^

Limitations of this study include its retrospective, observational design. As such, it is possible that AEs may have been underreported and the criteria used to classify symptoms or events as part of an AE may have varied between physicians/institutions. Moreover, this study employed purposive sampling, which may not represent all physicians and/or all patients with I/P-risk aRCC. Lastly, although the representation of African-American/Black patients in this study is greater than clinical trials, the sample size when stratified by type of first-line regimen was relatively small.

In summary, this study among patients with I/P-risk aRCC showed that African-American/Black patients presented with more adverse baseline clinical characteristics and trended toward numerically lower rwORR and shorter median OS for NIVO + IPI and PEM + AXI than White patients. Future studies with greater sample size, longer follow-up, and adjustment for potentially confounding factors are needed to confirm findings and further understand racial differences in first-line therapies for aRCC. More importantly, biological signatures, such as immune signatures, are necessary to more effectively select patients for appropriate first-line therapies. Such insights could pave the way for targeted approaches to reduce disparities, increase equitable care, and improve patient outcomes.

## Supplementary material

Supplementary material is available at *The Oncologist* online.

oyae354_suppl_Supplementary_Table_S1

## Data Availability

The data that support the findings of this study are not openly available due to reasons of sensitivity and privacy of the data sources.
