# Supplementary material for: Racial differences in real-world outcomes of first-line therapies for advanced renal cell carcinoma
Source: Oncologist. 2024 Dec 19;30(7):oyae354. doi: 10.1093/oncolo/oyae354 (PMC12311295; doi:10.1093/oncolo/oyae354)
Supplement: oyae354_suppl_Supplementary_Table_S1 [file oyae354_suppl_supplementary_table_s1.docx]

**Supplementary materials**

**Supplementary Table S1.** Characteristics of select AEs during first-line treatment stratified by race and first-line treatment regimen among patients with I/P-risk aRCC

|  | Overall AEs (N=121) | | | | 1L NIVO+IPI AEs (n=74) | | | 1L PEM+AXI AEs (n=28) | | | | 1L single agent TKI AEs (n=19) | | | |  |
| --- | --- | --- | --- | --- | --- | --- | --- | --- | --- | --- | --- | --- | --- | --- | --- | --- |
|  | Number of AEs-African American/Black | | Number of AEs-White | | Number of AEs-African American/Black | | Number of AEs-White | Number of AEs-African American/Black | | Number of AEs-White | | Number of AEs-African American/Black | | Number of AEs-White | |  |
|  | n=20 | | n=101 | | n=12 | | n=62 | n=8 | | n=20 | | n=0 | | n=19 | |  |
| **AE reported during 1L, n (%) ^1^** |  |  |  |  |  |  |  |  |  |  |  |  |  |  |  |  |
| Rash | 7 (35.0) | | 28 (27.7) | | 5 (41.7) | | 21 (33.9) | 2 (25.0) | | 4 (20.0) | | 0 (0.0) | | 3 (15.8) | |  |
| Thyroid abnormality | 6 (30.0) | | 24 (23.8) | | 3 (25.0) | | 18 (29.0) | 3 (37.5) | | 5 (25.0) | | 0 (0.0) | | 1 (5.3) | |  |
| Colitis | 1 (5.0) | | 17 (16.8) | | 1 (8.3) | | 9 (14.5) | 0 (0.0) | | 4 (20.0) | | 0 (0.0) | | 4 (21.1) | |  |
| ALT increased | 1 (5.0) | | 9 (8.9) | | 0 (0.0) | | 2 (3.2) | 1 (12.5) | | 4 (20.0) | | 0 (0.0) | | 3 (15.8) | |  |
| AST increased | 1 (5.0) | | 5 (5.0) | | 0 (0.0) | | 2 (3.2) | 1 (12.5) | | 1 (5.0) | | 0 (0.0) | | 2 (10.5) | |  |
| Arthralgia/arthritis | 1 (5.0) | | 5 (5.0) | | 0 (0.0) | | 4 (6.5) | 1 (12.5) | | 1 (5.0) | | 0 (0.0) | | 0 (0.0) | |  |
| Pneumonitis | 1 (5.0) | | 4 (4.0) | | 1 (8.3) | | 1 (1.6) | 0 (0.0) | | 0 (0.0) | | 0 (0.0) | | 3 (15.8) | |  |
| Hepatitis | 1 (5.0) | | 5 (5.0) | | 1 (8.3) | | 2 (3.2) | 0 (0.0) | | 1 (5.0) | | 0 (0.0) | | 2 (10.5) | |  |
| Myocarditis | 0 (0.0) | | 1 (1.0) | | 0 (0.0) | | 1 (1.6) | 0 (0.0) | | 0 (0.0) | | 0 (0.0) | | 0 (0.0) | |  |
| Other | 1 (5.0) | | 3 (3.0) | | 1 (8.3) | | 2 (3.2) | 0 (0.0) | | 0 (0.0) | | 0 (0.0) | | 1 (5.3) | |  |
| **Highest grade of AE experienced during 1L, n (%)** |  |  |  |  |  |  |  |  |  |  |  |  |  |  |  |  |
| 1 | 8 (40.0) | | 39 (38.6) | | 4 (33.3) | | 20 (32.3) | 4 (50.0) | | 11 (55.0) | | 0 (0.0) | | 8 (42.1) | |  |
| 2 | 9 (45.0) | | 38 (37.6) | | 5 (41.7) | | 25 (40.3) | 4 (50.0) | | 6 (30.0) | | 0 (0.0) | | 7 (36.8) | |  |
| 3 | 2 (10.0) | | 15 (14.9) | | 2 (16.7) | | 11 (17.7) | 0 (0.0) | | 1 (5.0) | | 0 (0.0) | | 3 (15.8) | |  |
| 4 | 1 (5.0) | | 3 (3.0) | | 1 (8.3) | | 2 (3.2) | 0 (0.0) | | 1 (5.0) | | 0 (0.0) | | 0 (0.0) | |  |
| Unknown | 0 (0.0) | | 6 (5.9) | | 0 (0.0) | | 4 (6.5) | 0 (0.0) | | 1 (5.0) | | 0 (0.0) | | 1 (5.3) | |  |
| **Action taken for AE experienced during 1L, n (%)†** |  |  |  |  |  |  |  |  |  |  |  |  |  |  |  |  |
| No action taken | 16 (80.0) | | 74 (73.3) | | 8 (66.7) | | 47 (75.8) | 8 (100.0) | | 17 (85.0) | | 0 (0.0) | | 10 (52.6) | |  |
| Therapy delayed/slowed | 2 (10.0) | | 12 (11.9) | | 2 (16.7) | | 9 (14.5) | 0 (0.0) | | 0 (0.0) | | 0 (0.0) | | 3 (15.8) | |  |
| Therapy stopped | 2 (10.0) | | 10 (9.9) | | 2 (16.7) | | 6 (9.7) | 0 (0.0) | | 2 (10.0) | | 0 (0.0) | | 2 (10.5) | |  |
| Therapy dose change | 0 (0.0) | | 5 (5.0) | | 0 (0.0) | | 0 (0.0) | 0 (0.0) | | 1 (5.0) | | 0 (0.0) | | 4 (21.1) | |  |
| **Treatment/intervention for reported AEs, n (%)** |  |  |  |  |  |  |  |  |  |  |  |  |  |  |  |  |
| Hospitalization | 2 (10.0) | | 6 (5.9) | | 2 (16.7) | | 3 (4.8) | 0 (0.0) | | 1 (5.0) | | 0 (0.0) | | 2 (10.5) | |  |
| ED admission | 2 (10.0) | | 1 (1.0) | | 2 (16.7) | | 1 (1.6) | 0 (0.0) | | 0 (0.0) | | 0 (0.0) | | 0 (0.0) | |  |
| Steroid use | 8 (40.0) | | 40 (39.6) | | 7 (58.3) | | 26 (41.9) | 1 (12.5) | | 5 (25.0) | | 0 (0.0) | | 9 (47.4) | |  |
| No treatment/intervention | 7 (35.0) | | 29 (28.7) | | 2 (16.7) | | 18 (29.0) | 5 (62.5) | | 5 (25.0) | | 0 (0.0) | | 6 (31.6) | |  |
| Other | 5 (25.0) | | 33 (32.7) | | 3 (25.0) | | 20 (32.3) | 2 (25.0) | | 9 (45.0) | | 0 (0.0) | | 4 (21.1) | |  |
| Unknown | 0 (0.0) | | 2 (2.0) | | 0 (0.0) | | 0 (0.0) | 0 (0.0) | | 2 (10.0) | | 0 (0.0) | | 0 (0.0) | |  |
| Key: 1L - first line; AE – adverse event; ALT - alanine transaminase; AST - aspartate aminotransferase; CI – confidence interval; ED – emergency department; min - minimum; max - maximum; p25-p75 - 25th and 75th percentiles; TKI - tyrosine kinase inhibitor. | | | | | | | | | | | | | | | | |
| Percentages may not sum to 100.0% (or specified column total) due to rounding | | | | | | | | | | | | | | | | |
| ^1^Selected AE/toxicities were self-reported by provider; provider may have indicated up to 5 AEs/ toxicities during 1L. If a patient had any AEs during the first phase of data collection, additional AEs were not assessed in extended follow-up data collection; extended follow-up only collected data on AEs for patients with no AEs reported during the first phase of data collection. | | | | | | | | | | | | | | | | |
| † Categories of response not mutually exclusive; total may sum to more than column total. | | | | | | | | | | | | | | | | |
